# Pharmacometabolomics study identifies circulating spermidine and tryptophan as potential biomarkers associated with the complete pathological response to trastuzumab-paclitaxel neoadjuvant therapy in HER-2 positive breast cancer

**DOI:** 10.18632/oncotarget.9489

**Published:** 2016-05-19

**Authors:** Gianmaria Miolo, Elena Muraro, Donatella Caruso, Diana Crivellari, Anthony Ash, Simona Scalone, Davide Lombardi, Flavio Rizzolio, Antonio Giordano, Giuseppe Corona

**Affiliations:** ^1^ Department of Medical Oncology, IRCCS-National Cancer Institute, Aviano, Italy; ^2^ Department of Translational Research, IRCCS-National Cancer Institute, Aviano, Italy; ^3^ Department of Pharmacological and Bimolecular Science, University of Milan, Milan, Italy; ^4^ Department of Biological Chemistry, Norwich Research Park, Norwich, United Kingdom; ^5^ Sbarro Institute for Cancer Research and Molecular Medicine, Center for Biotechnology, College of Science and Technology, Temple University, Philadelphia, PA, USA

**Keywords:** pharmacometabolomics, pharmacometabonomics, metabolomics, breast, cancer

## Abstract

Defining biomarkers that predict therapeutic effects and adverse events is a crucial mandate to guide patient selection for personalized cancer treatments. In the present study, we applied a pharmacometabolomics approach to identify biomarkers potentially associated with pathological complete response to trastuzumab-paclitaxel neoadjuvant therapy in HER-2 positive breast cancer patients. Based on histological response the 34 patients enrolled in the study were subdivided into two groups: good responders (*n* = 15) and poor responders (*n* = 19). The pre-treatment serum targeted metabolomics profile of all patients were analyzed by liquid chromatography tandem mass spectrometry and the differences in the metabolomics profile between the two groups was investigated by multivariate partial least squares discrimination analysis. The most relevant metabolites that differentiate the two groups of patients were spermidine and tryptophan. The Good responders showed higher levels of spermidine and lower amounts of tryptophan compared with the poor responders (*p* < 0.001, *q* < 0.05). The serum level of these two metabolites identified patients who achieved a pathological complete response with a sensitivity of 90% [0.79–1.00] and a specificity of 0.87% [0.67–1.00]. These preliminary results support the role played by the individual patients' metabolism in determining the response to cancer treatments and may be a useful tool to select patients that are more likely to benefit from the trastuzumab-paclitaxel treatment.

## INTRODUCTION

The introduction of trastuzumab revolutionized the treatment of HER-2-positive breast cancer (BC) and now represents the gold standard drug used alongside other pharmacological regimens both in adjuvant and neoadjuvant settings [1–4]. The latter approach may be able to reduce the tumour mass of locally advanced BC, decrease the subclinical micro-metastatic disease and improve the rates of breast-conserving surgeries [1, 5]. In this setting, the induction of a pathological complete response (pCR), i.e. the absence of cancer cells in breast tissue after therapy, is associated with improved disease free survival (DFS), overall survival (OS) and reduced relapse rates [6, 7]. Even if the addition of trastuzumab strongly increased the pCR rates in HER-2 positive BC patients [8] there is still a significant subgroup of patients that does not benefit from this treatment [9]. For this reason, the identification of specific biomarkers that are able to predict an unfavourable response to trastuzumab-based neoadjuvant treatment could be clinically useful in order to select more effective treatments.

The survey for biomarkers, managed both through retrospective clinical analysis and gene expression studies [10] proved to be very challenging, showing that also the level of HER-2 expression, the most obvious candidate biomarker for HER-2 targeting therapy, resulted to be surprisingly ambiguous [11–13]. The multifaceted mode of action of trastuzumab may be responsible for the difficulty in identifying, predictive biomarkers since as monoclonal antibody trastuzumab acts also through immune-mediated mechanisms that involve host immune cells [14]. The required contribution of host effectors suggests a direct involvement of individuals' endogenous biochemical factors, which could thus represent an innovative and complementary front for predictive biomarkers research. Moreover, other recently proposed surrogate markers of response to neoadjuvant treatment are based on patients' characteristics such as the body mass index (BMI). Indeed, a higher BMI was found associated with lower rate of pCR and a detrimental impact on survival [15]. This contributes to retain that the host response to the tumour is perhaps equally important to determining the pharmacological effect, which can be highlighted by an individual's specific biochemical signature.

Pharmacometabolomics is an innovative “omic” methodology that is able to access patients' biochemical status and is therefore useful to understand the host's response to drug treatments. This new approach is based on the identification of individuals' metabolomic profiles, which represent a large repertoire of metabolites that may reflect the complex interactions among gene expression, protein expression, physio-pathological conditions, age, gut microbiome, and the environment better than other “omic” profiles. Consequently, the metabolomics profile is more closely associated to a patient's pharmacological phenotype and could be more informative than genomic and proteomic data clarifying better the mechanisms of inter-patient variability to drug therapy [16–19]. The potential for pharmacometabolomics has already been demonstrated for different classes of drugs in preclinical investigations, as well as in clinical pharmacological settings [20–25]. A few investigations have been applied to the pharmacological treatment of BCs. In particular, two previous investigations analysed the predictive value of basal serum metabolites on the outcome of BC patients in the metastatic and neoadjuvant settings [26, 27] but their results were contradictory. One of these studies consisted of a pilot multicentric investigation that attempted to correlate the serum metabolomics profile with patient outcome and treatments toxicity [26]. However, this study failed to identify any correlations between the patients' metabolomics profile and the therapies effect. More recently, a metabolomics investigation found that pCR correlated with changes in serum level of methionine, glutamine and linoleic acid [27]. However, the histological heterogeneity of the tumours and the different treatments administered limited the data interpretation for both these studies.

The present study was designed to explore the potential use of a pharmacometabolomics approach as a means to identify pre-treatment serum metabolite biomarkers associated with the pCR in a homogeneous monocenter cohort of HER-2 positive BC patients after neoadjuvant trastuzumab-paclitaxel treatment. We found that patients with lower serum concentrations of tryptophan and higher concentrations of spermidine responded more successfully to the treatment. This underlines the role that the individual's metabolic trait may have on determining the pharmacological outcome of cancer therapy.

## RESULTS

### Patient characteristics

The demographic and pathological characteristics of the patients investigated are reported in Table 1. Patients were divided into two groups according to their histological response to the trastuzumab-paclitaxel neoadjuvant treatment. The good responders (GR) (*n* = 15), who achieved pCR and the poor responders (PR) (*n* = 19), who achieved only a partial pathological response where residual disease was still revealed after the neoadjuvant treatment. The two groups did not differ significantly by age, BMI, tumour stage, grade and hormonal receptor expression.

**Table 1:** Patients' characteristics

Parameters	*GR*	*PR*	*p*
Patients			
34	15	19	
Age (years)			NS^#^
Median (range)	42 (28–58)	49 (23–70)	
BMI (Kg/m2)			NS^#^
Mean ± SD	24.1 ± 4.9	25.8 ± 5.6	
Stage			NS^#^
IIA	2	1	
IIB	8	16	
IIIA	5	2	
Grade			NS^§^
G2	2	3	
G3	9	15	
Gx	4	1	
ER/PgR			NS^§^
neg/neg	7	8	
pos/neg	4	3	
pos/pos	4	7	
neg/pos	0	1	

# unpaired *t*-Test,

§ Chi-Square test.

BMI Body mass index, ER:Estrogen receptors, PgR: progesterone receptors.

In both groups approximately half of the patients had hormone receptor positive tumours. Although not statistically significant at diagnosis, the GR group was characterized by a higher frequency of stage III tumours, which is generally indicative of a more aggressive phenotype with a relatively poor prognosis.

### Metabolomics data analysis and biomarker identification

**Figure 1:**
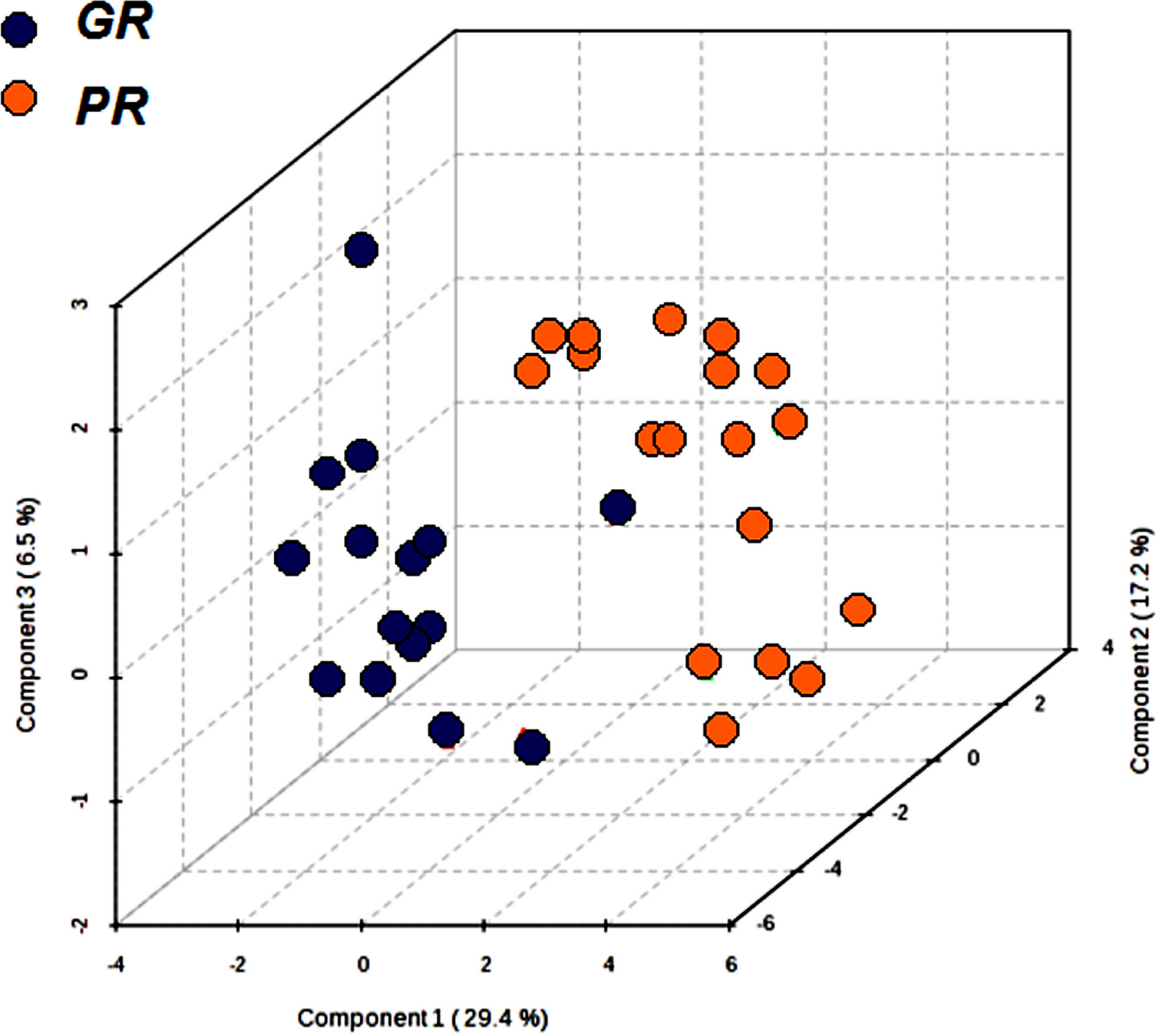
Partial least squares discrimination analysis (PLS-DA) graph used to distinguish the metabolomics profile of the two groups GR (*n* = 15) and PR (*n* = 19) Each point corresponds to the metabolomics profile of a patient.

The pre-treatment serum targeted metabolomics profile data, determined by a validated LC-MS/MS, were analysed using supervisor partial least squares discrimination analysis (PLS-DA). This analysis was carried out to investigate if differences in the quantitative metabolomics profiles of the patients were able to distinguish the GR from the PR group. The results of this multi-parametric approach are summarized in the PLS-DA graph (Figure 1), where each point corresponds to the metabolite profile of each patient. From the quantitative metabolomics data, the GR group had a spatial distribution that was significantly different from that of PR group. The PSA-DA model was further refined to eliminate potential noise by focusing on the metabolites that showed a significant difference among the two groups of patients. The internal cross validation of the refined PLS-DA model used in this study demonstrated good modelling and predictive capabilities (85% accuracy, good R^2^ (0.73) and Q^2^ (0.57). The values and permutation testing did not reveal any significant (*p* < 0.003) potential for over-fitting the model.

**Figure 2:**
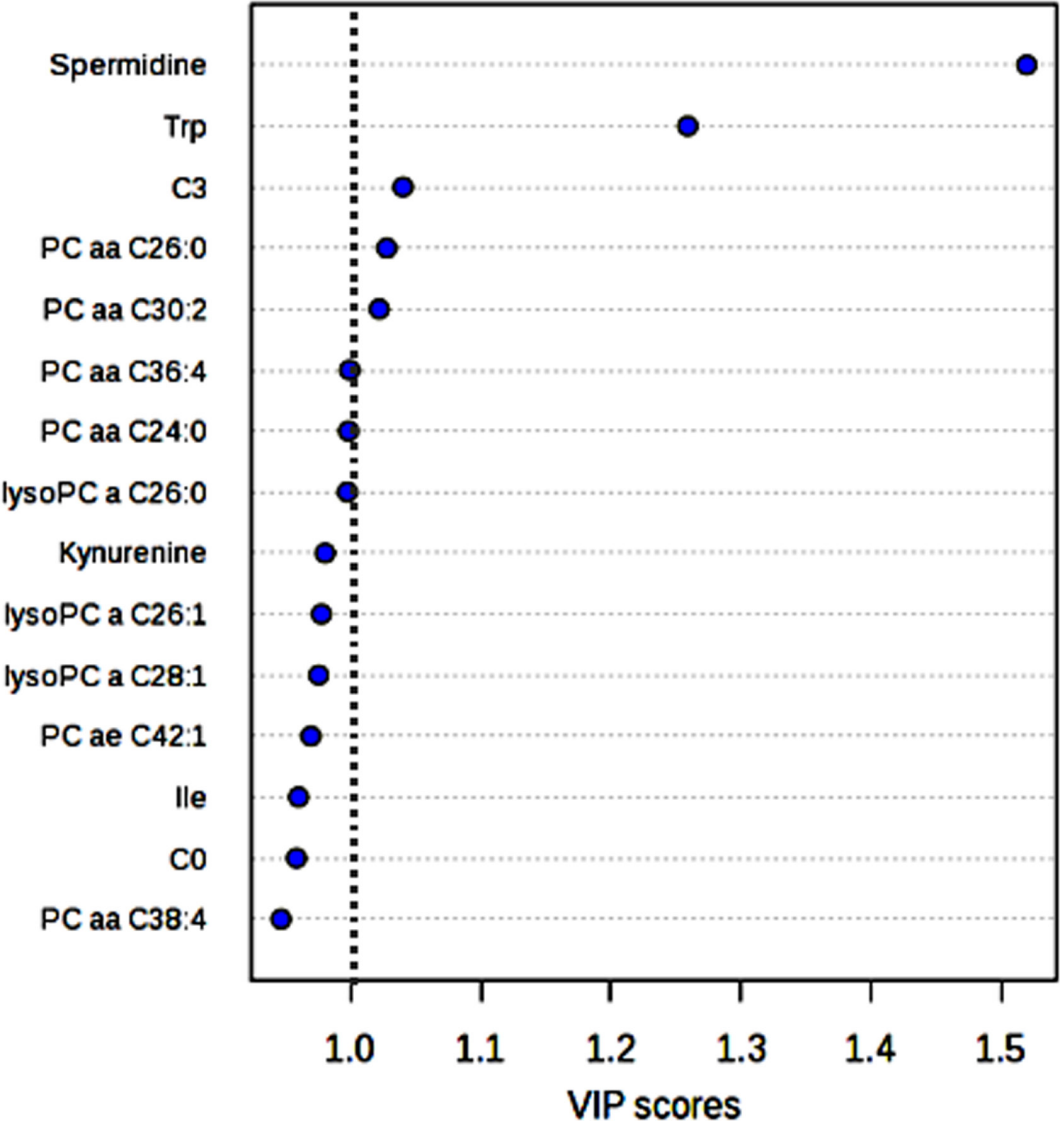
Metabolites that produce the largest contribution in discriminating between GR and PR groups in the PLS-DA model, relative to the VIP score

**Figure 3:**
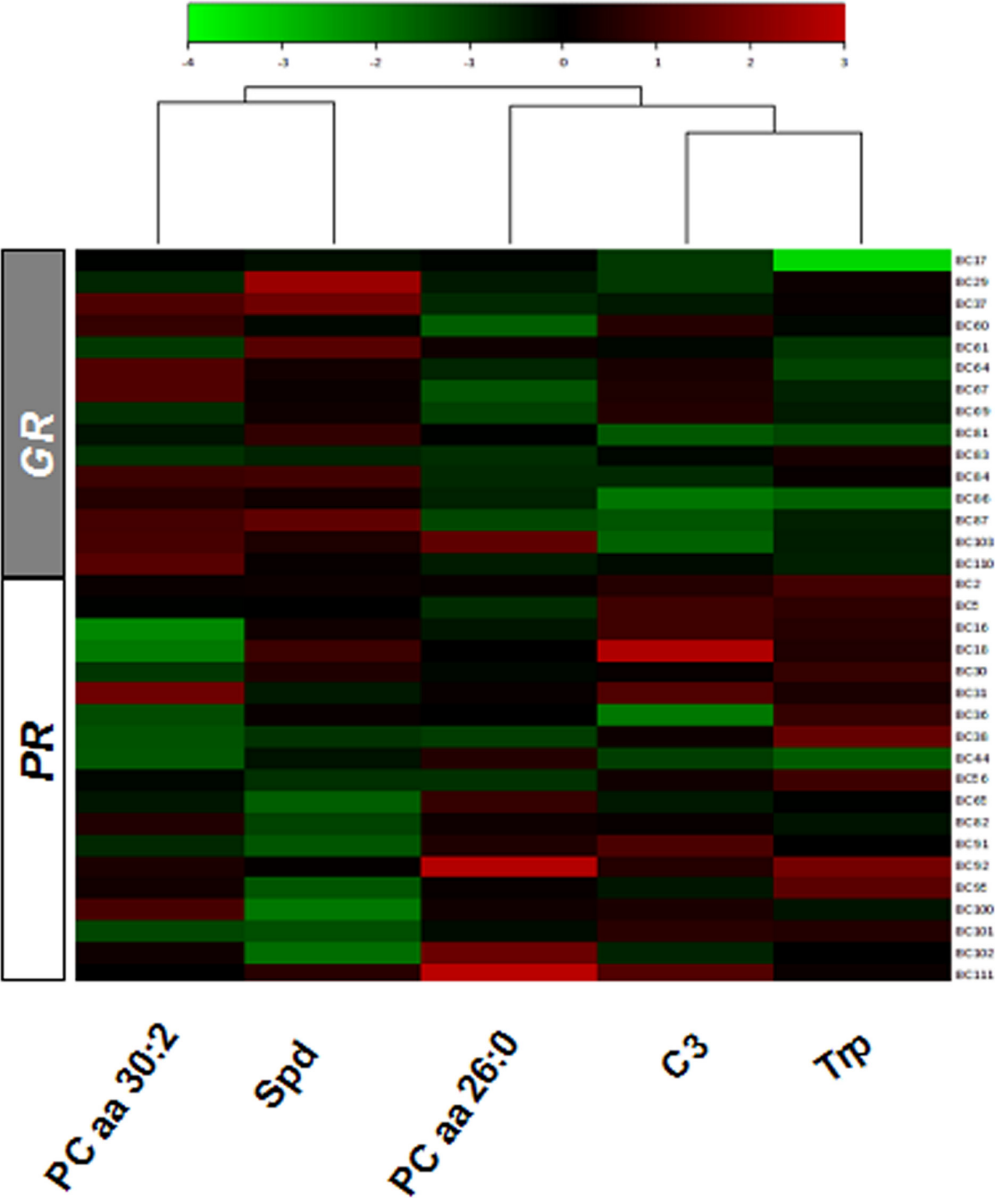
Heatmap of the relative concentration of five metabolites with VIP > 1 in the serum of the GR and PR groups of patients according to the response to trastuzumab-paclitaxel treatment

**Figure 4:**
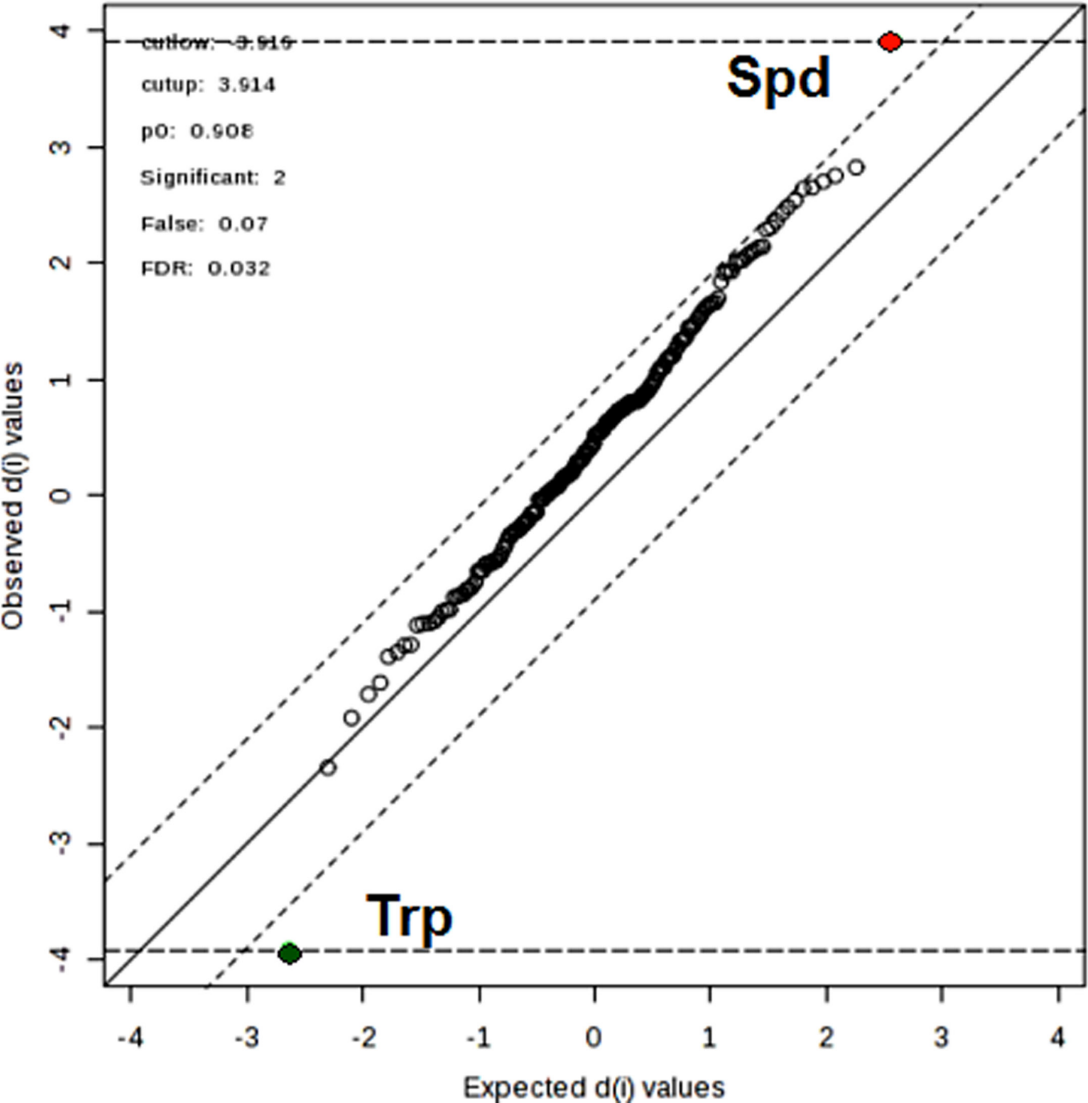
Significance analysis of microarray plot sheet output (SAM) under the four sets of criteria: cutup = 3.916, cutlow = 3.914; false=0.07; FDR = 0.032; which are indicated at the upper right corner The red, green, and black dots represent upregulated, downregulated, and insignificant metabolites.

In order to select the metabolites that showed a significant difference between the GR and PR metabolomic profiles a variable influence on the projection (VIP) parameter was used. The most relevant metabolites (VIP > 1) were: spermidine (Spd), tryptophan (Trp), propylcarnitine (C3) and the two phosphatidylcholine diacyl phospholipids (PC aa) PC aa C26:0 and PC aa C30:2 (Figure 2). The relative concentration distributions of such metabolites between the two groups of patients can be visualized in a heatmap (Figure 3). Among the five relevant metabolites (Spd; Trp; C3; PCaa C26:0 and PC aa C30:2), the difference in Spd concentration levels resulted the most clearly differentiated between the two groups. Furthermore, when an alternative statistical analysis was performed such as the Significance Analysis of Microarray (SAM) to account for potential False Discovery Rates (FDR), only Spd and Trp showed significant differences between the two groups of patients (Figure 4). The mean ± SD serum concentration of bioactive amine Spd in the GR group was significantly higher, 0.15 ± 0.06 μM *vs* 0.09 ± 0.032 μM (*p* < 0.001, *q* < 0.05), relative to the PR group. Whilst, the level of Trp was significantly lower in the GR group 61.19 ± 8.46 μM *vs* 73.82 ± 9.23 μM (*p* = 0.001, *q* < 0.05) relative to the PR group. Consequently, the anabolic and catabolic routes involved in the metabolism of Spd and Trp between the two groups of patients was further investigated. This analysis was carried out using the available quantitative metabolomics profile data, to explore any other associated metabolic alterations that could be potentially useful in the development of a predictive model for pCR after neoadjuvant trastuzumab-paclitaxel treatment.

### Spermidine and tryptophan metabolic pathways

**Figure 5:**
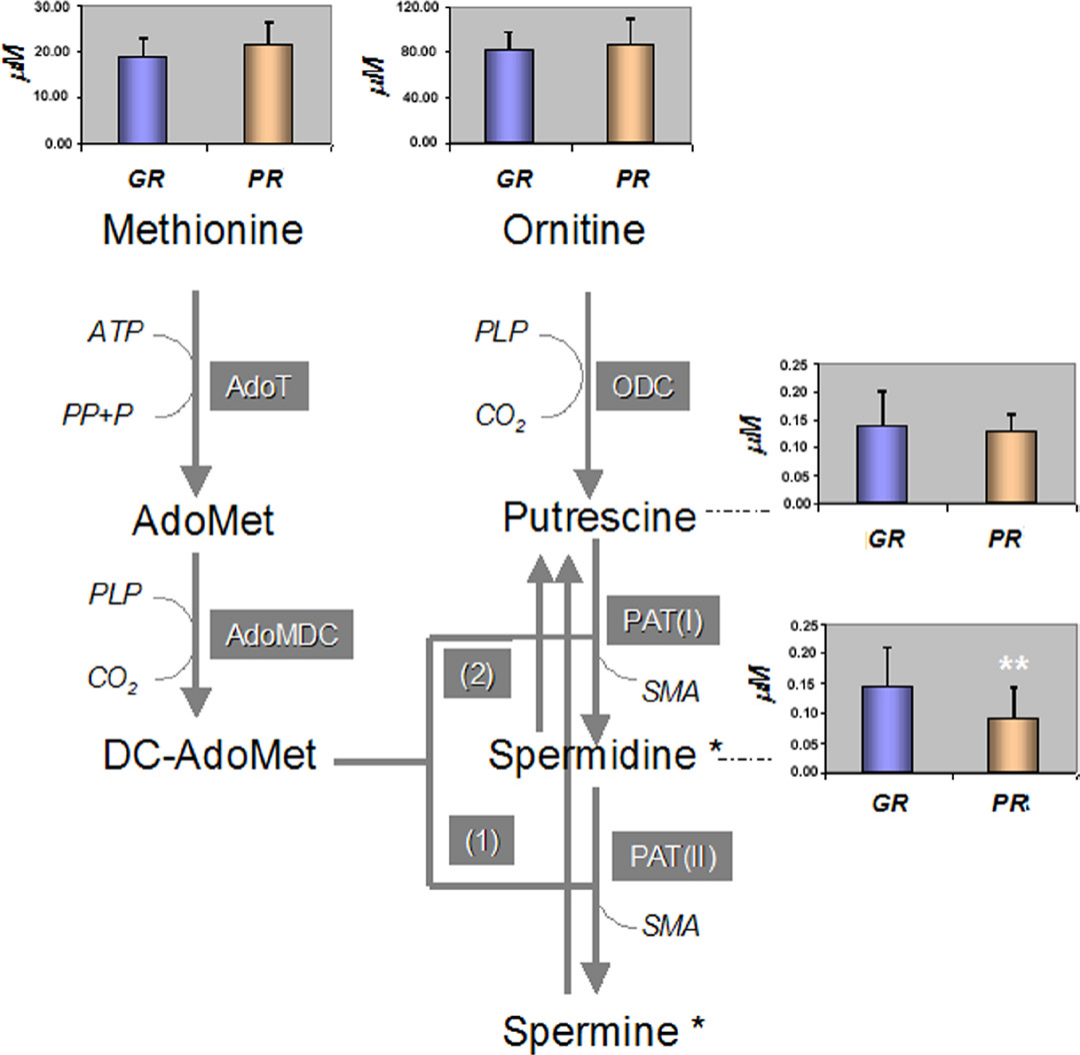
Polyamine biosyntethic pathway and the mean concentration [μM] of metabolites among the GR and PR groups of patients A *p* value of *p < 0.05 and **p < 0.001 was considered statistically significant. AdoMet: S-Adenosyl-Methionine, DC-AdoMet: Decarboxylated S-Adenosyl-Methionine, AdoT: S-AdenosylTransferase, AdoMDC: S-Adenosyl-Methionine Decarboxylase, ODC: Ornitine Decarboxylase, PAT(I and II): PropylAminoTransferase-1 and 2, ATP: AdenosylTriphosphate. SMA: S-MethylAdenosyl. PLP: Pyridoxal-5′-Phosphate, (*) Spermine and Spermidine can be inter converted to Putrescine in two steps catalized by the (*1*) Spermine/Spermidine-N-1-AcetylTransferase (SSAT); (*2*) Polyamine Oxidase (PO).

The Spd is a metabolite derived from the catabolic process of ornitine (Orn) to putrescine (Put) that requires methionine (Met) activation for its synthesis (Figure 5).

The quantitative data from the metabolomics profile indicated that between the GR and PR groups there were no significant differences in the serum levels of the Orn and Met precursors: 81 ± 16 μM *vs* 87 ± 22 μM for Orn and 18.87 ± 4.08 μM *vs* 21 ± 5 μM for Met, respectively. In addition, the serum level of Put showed no significant differences between the two groups of patients: GR 0.14 ± 0.05 μM vs PR 0.13 ± 0.05 μM. Spermine, which is also involved in the pathway of bio-amine synthesis, was lower than the limit of detection for all patients investigated. The GR group characterized by a higher concentration of Spd as compared with the PR group, showed a significantly lower Orn/Spd ratio: 610 ± 205 *vs* 1107 ± 534 (*p* < 0.05) and a significantly lower Put/Spd ratio 1.0 ± 0.4 *vs* 1.6 ± 0.8 (*p* < 0.05).

**Figure 6:**
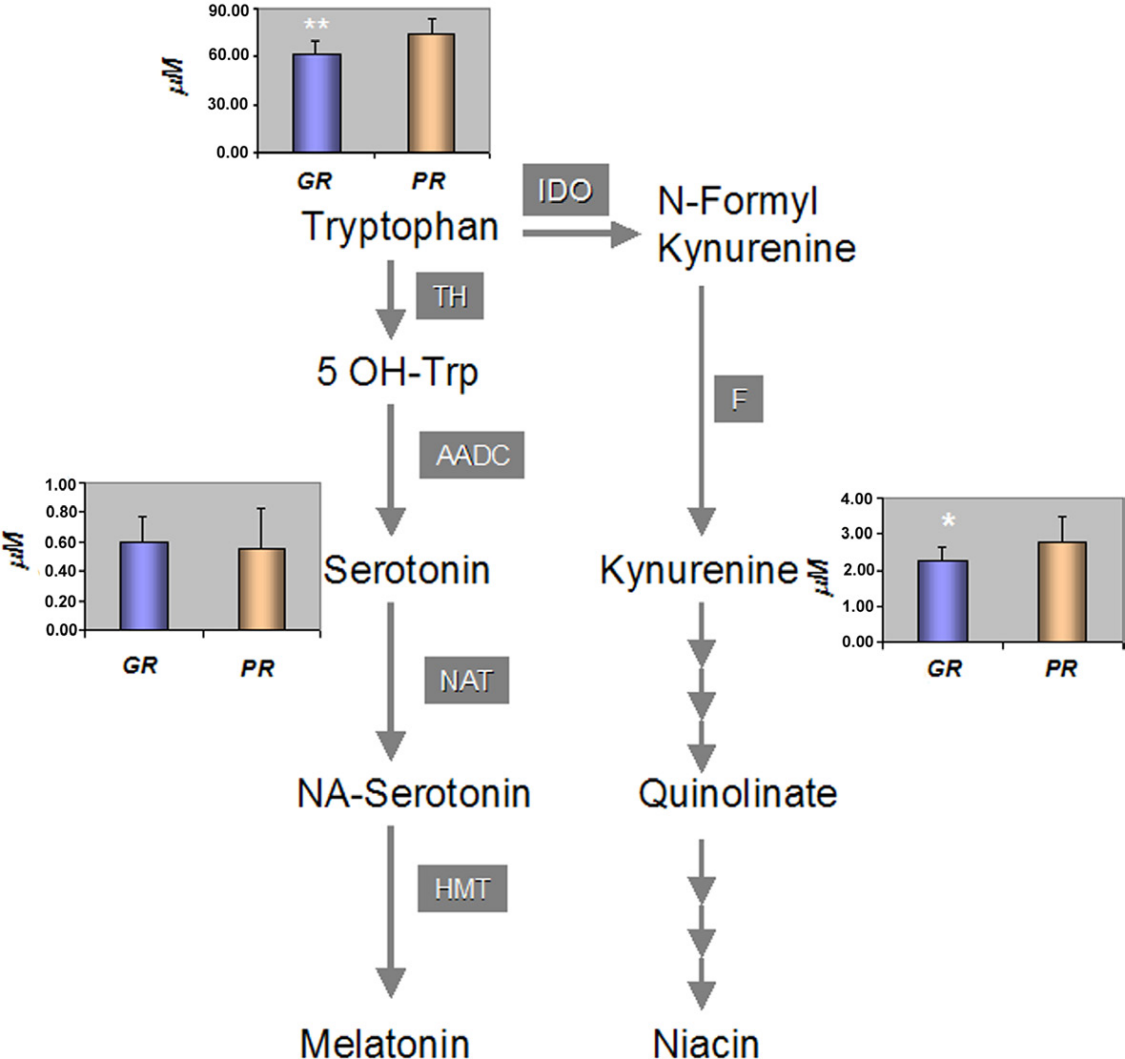
Tryptophan catabolic pathway and the mean concentration [μM] of metabolites among the GR and PR groups of patients A *p* value of *p < 0.05 and **p < 0.001 was considered statistically significant. TH: Tryptophan Hydroxylase, AADC: Aromatic Amino Acid Decarboxylase, NAT: N-Acetyl Transferase, HMT: 5-Hydroxylindole-O-MethylTransferase, Indoleamine-2, 3 dioxygenase, F: Formamidase.

The Trp represents an essential amino acid that provides a building block to a vast array of proteins. It also acts as biochemical precursor for the synthesis of niacin cofactor, through pathways of kynurenine (Kyn) and for the synthesis of melatonin from serotonin (Set) via the methoxyindoles pathway (Figure 6). Within the Kyn pathway, the low serum level of Trp observed in the GR group was associated with a significantly (*p* < 0.05) lower level of the intermediate Kyn, 2.2 ± 0.4 *vs* 2.8 ± 0.7 μM, compared to the PR group. However, the ratio between Trp and Kyn showed no significant differences between two groups (27 ± 4 vs 28 ± 7). In addition, the mean serum concentrations of Set (via the methoxyindoles pathway) also showed no significant differences between the two groups, GR 0.60 ± 0.16 μM *vs* PR 0.55 ± 0.27 μM while the mean value of the Trp/Set ratio was significantly lower in the GR group compared with the PR group: 108 ± 29 *vs* 159 ± 70, (*p* < 0.05) (Figure 6).

### Receiver operating characteristic curve

**Figure 7:**
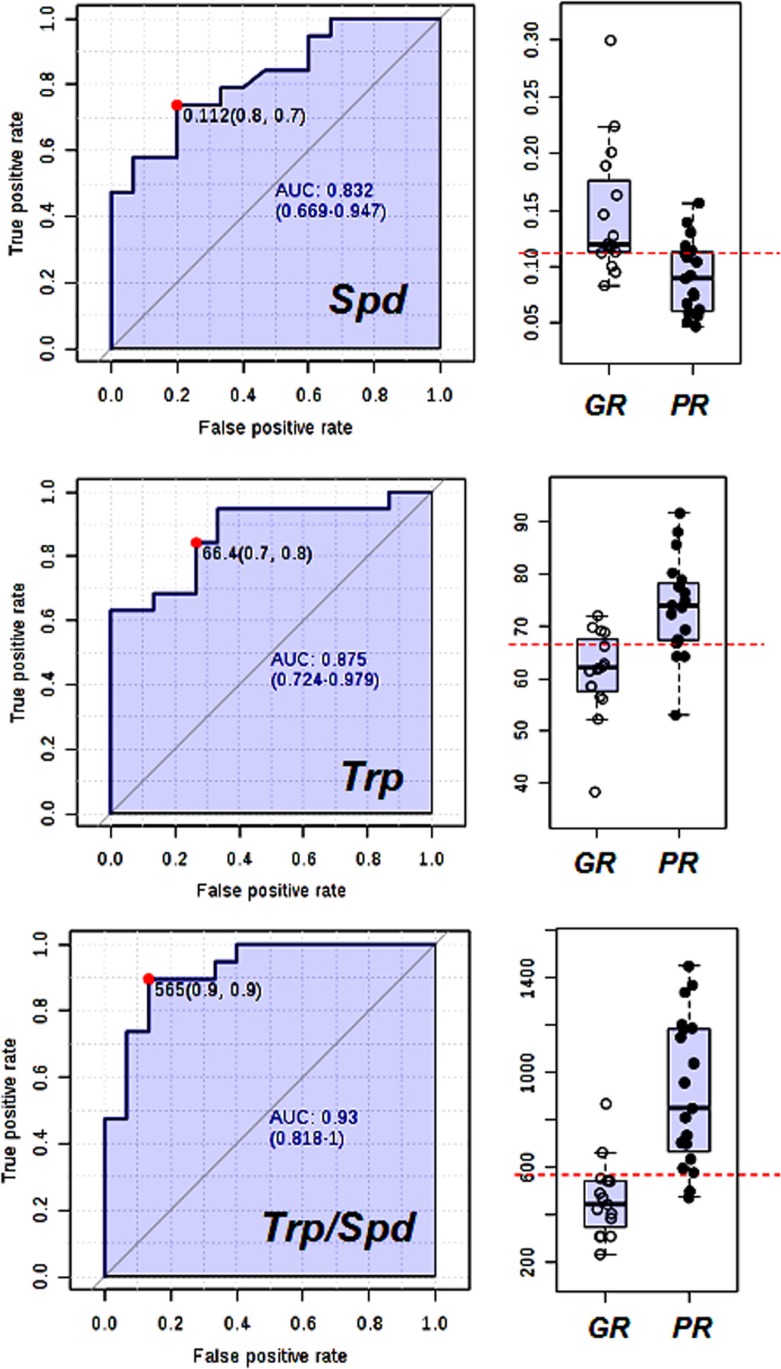
Receiving operating and scatter plots curve for (a) Spd, (b) Trp and (c) Trp/Spd ratio Area under curve plots (95% confidence interval in brackets).

To demonstrate the diagnostic value of the two most relevant serum metabolites, a receiver operating characteristic (ROC) curve was applied to the data. This helped to evaluate the predictive power of the two metabolites in identifying patients that would best benefit from the neoadjuvant treatment. The ROC curves were produced using the serum concentration of Spd and Trp both independently and combined. The ROC curve data was obtained by plotting the sensitivity and specificity values for each concentration of biomarkers and the threshold value for each metabolite was determined by searching for those that yielded both high sensitivity and specificity. The serum levels of the selected metabolic biomarkers Spd and Trp can be influenced by confounding effects such as age, BMI, and tumour stage. For this reason, the effect of these covariates on the sensitivity and specificity of ROC model, based on Trp and Spd metabolites, was investigated. The results of this investigation clearly showed that age, BMI and tumour stage had no effect on the predictive diagnostic power of Spd and Trp, indicating that these two metabolic biomarkers can be considered as independent factors for the response to neoadjuvant treatment (Figure S1 supplementary data). In Figure 7, the ROC curves for Spd, Trp, and Trp/Spd ratio with the associated area under curve (AUC) are reported. Considering Spd and Trp as single biomarkers, the AUC [95% confidence interval] was 0.83 [0.67–0.95] and 0.87 [0.72–0.98] respectively, whilst the AUC value increased to 0.93 [0.82–1.00] when the Trp/Spd ratio was used. Therefore, using a threshold value of 565 for the Trp/Spd ratio it was possible to distinguish patients achieving a pCR after neoadjuvant treatment with high sensitivity 0.90 [0.79–1.00] and high specificity 0.87 [0.67–1.00].

## DISCUSSION

In this pharmacometabolomics investigation, we applied a targeted metabolomics approach to assess the diagnostic potential of endogenous pre-treatment serum metabolites profile to predict the response to trastuzumab-paclitaxel treatment in a neoadjuvant setting. Unlike previous clinical metabolomics investigations [26, 27] this study focused on a homogeneous population of BC patients. We have observed that patients with higher serum concentrations of Spd and lower concentrations of Trp would be more likely to benefit from trastuzumab–paclitaxel neoadjuvant treatment. The Spd, spermine, and Put are essential ubiquitary cellular polyamines essentially constituted of organic polycations having variable hydrocarbon chains and two or more primary amino groups [28]. These chemical features are responsible for their essential role in DNA and RNA metabolism, maintenance of chromatin structure, regulation of specific gene expression, ion channel modulation and membrane stability. The key step in the production of polyamines is regulated by Orn decarboxylase (ODC), which is required for the production of Put. Subsequently, propylamino-transferase catalyzes the transfer of Put of the aminopropyl group from decarboxylated S-adenosylmethionine to form Spd and spermine, respectively (Figure 5). This process is important because polyamines are essential for growth and differentiation of cells. In general, high concentrations of polyamines are found in rapidly growing cells, while low concentrations are present in quiescent cells [29, 30]. An increased production is observed in many cancer tissues including BC [31]. However, the function of such high concentrations of polyamines in tumour cells is not clear. In a study addressed to evaluate the variation of polyamines in the pre and post-surgery serum of BC patients, no significant difference was reported [32]. This suggests that the circulatory level of polyamines is more likely to represent an individual's general physio-pathological condition. Thus, the difference in the Spd level between the GR and PR groups observed in this study could represent a host's metabolic feature not representative of the tumour's metabolic activity. In addition, the low level of serum Spd in the PR group appears not to be associated with differences in the level of Orn, Arg, Met and Put. Thus indicating that it may not be due to a shortage of the metabolite precursors. Moreover, the Orn/Spd and Put/Spd ratios, considered as surrogate parameters of Spd biosynthesis efficacy, were both lower in the GR group compared to PR patients. This suggests that a high synthetic rate of Spd could be a characteristic of patients that are more likely to achieve a pCR. However, a reduced catabolic clearance of Spd could also explain the higher concentration of Spd observed. Further investigation into the clearance of acetyl derivatives of polyamine [33] may give a more comprehensive explanation for the elevated Spd serum levels observed in the GR group.

It is possible that a high level of circulating Spd could be translated into a high concentration of polyamines in the tumour microenvironment that may boost cell replication, thus improving paclitaxel activity. This drug promotes the assembly of microtubules from tubulin dimers and stabilises them by preventing depolymerisation. This stability may cause the inhibition of the normal dynamic reorganisation of the microtubule network, which is essential for vital interphase and mitotic cellular functions. Since Spd has been reported to improve tubulin assembly [34], a high concentration of this polyamine may also have a synergistic effect on the stabilization of microtubules, thus potentially increasing paclitaxel cytotoxicity.

The other relevant metabolic features that characterised the GR group of patients was the lower level of Trp. This metabolite is required for both protein synthesis and other important metabolic functions [35]. Low Trp serum levels may depend on a reduced dietary intake or an increase in its catabolism. Two major non-protein metabolic routes use Trp as a precursor: (*a*) the methoxyindoles pathway, involved in the synthesis of Set and Melatonin; and (*b*), the Kyn pathway, responsible for the production of Kyn through Niacin synthesis. Low levels of Trp, due to an increase activity of the Kyn pathway, can suppress the immune system [36–38]. Furthermore, the induction of this pathway has been recently proposed as a potential mechanism activated by the tumour to escape immune surveillance [39; 40]. Therefore, the involvement of Trp in the modulation of the immune system could be relevant for the immune-mediated activity of trastuzumab. Trastuzumab confers cytostatic effects through direct interaction with HER-2 receptors expressed by cancer cells suppressing the HER-2 dependent signalling pathway [41]. However, other mechanisms of action involving immune cells have been proposed for trastuzumab: the antibody-dependent cellular cytotoxicity (ADCC), which involves especially Natural Killer cells, and the activation of HER-2-specific cytotoxic T-cells (CD8^+^) [14, 42]. Thus, the level of tryptophan and its catabolic product in the tumour microenvironment may contribute to modulate the immunocytoxic side activity induced by Trastuzumab.

When the levels of Kyn and Set were compared between the two groups, the GR showed lower levels of Kyn, suggesting that the lower amount of Trp observed was not due to an increased catabolism. Both groups had the same Kyn catabolic activity as demonstrated by the lack of significant differences in the Trp/Kyn ratio. Conversely, an increase in Trp anabolism via the Set pathway could also be potentially relevant in the PR group as reported by the lower Trp/Set ratio observed. The low availability of Trp may be associated with a reduced Kyn biosynthesis, which acts as inhibitor of the immune response and thus may have a positive impact on the trastuzumab activity, contributing to the induction of a pCR in the GR group.

Finally and more importantly, the serum pre-treatment levels of both Spd and Trp can be used as predictive markers to distinguish patients who are more likely to achieve pCR after trastuzumab-paclitaxel neoadjuvant treatment. In fact, when the two metabolites were combined in the Trp/Spd ratio, the inter-patient variation was reduced and the diagnostic power reached a high sensitivity of 90% with a specificity of 87% (Figure 7).

In conclusion, this exploratory study demonstrated the importance of highlighting endogenous metabolic pathways that could favour the efficacy of neoadjuvant trastuzumab-paclitaxel treatment in HER-2 positive BC patients. The pharmacometabolomics approach adopted identified two major metabolic biomarkers represented by, Spd and Trp, that could potentially have a pivotal role in determining the efficacy of a trastuzumab-paclitaxel drug combination. Such an individual metabolic signature could be translated into a non-invasive diagnostic tool to select patients more likely to benefit from neoadjuvant treatment. Although these preliminary results are very encouraging, they are based upon a relatively small sample size and additional studies with an increased number of patients will be needed to confirm and clinically validate these exciting observations.

## MATERIALS AND METHODS

### Patients' population

Thirty-four patients were enrolled in this investigation. All patients had histologically confirmed locally advanced HER-2 positive BC (UICC stage II–III, non-inflammatory); patients were not previously treated with chemotherapy or hormonal therapy, an age ≤ 70 years, an Eastern Cooperative Oncology Group performance status ≤ 1, adequate hepatic, renal and bone marrow function. No CNS metastases, prior myocardial infarction or uncontrolled arrhythmia, angina pectoris, psychiatric syndromes or other serious medical conditions were present. Furthermore, no other concurrent malignancy, apart from non-melanoma skin cancer, or *in situ* cervix carcinoma was allowed.

Baseline evaluation included a physical examination, diagnostic breast imaging, abdominal ultrasound and bone scintigraphy. This study was carried out according to the ethical principles of the Declaration of Helsinki and approved by the Internal Institutional ethics committee. All patients enrolled in the investigation gave written informed consent.

### Clinical samples

After a period of overnight fasting, each patient provided a 5 ml blood sample on the first day of admission, prior to receiving the neoadjuvant therapy. The blood was drawn in the morning between 8 and 10 a.m. to avoid variation due to circadian rhythms. Blood was collected in BD Vacutainer^®^ tubes coated with silica particles as a clot activator. The blood was inverted five times and allowed 30 minutes clotting time and was then centrifuged at 2000g for 10 min at 4°C. Serum was then aliquot and frozen at −80°C until required for targeted metabolomics analysis.

### Study design

All patients enrolled in this study received trastuzumab: loading dose 4 mg/kg intravenously, then 2 mg/kg weekly and concomitant weekly paclitaxel (80 mg/m^2^) for 6 cycles. After neoadjuvant completion, patients underwent primary surgery, which consisted in a mastectomy or a quadrantectomy conservative treatment as well as axillary node dissection. In accordance to the histological response, the patients were classified in two groups: Good and Poor responders. The good responders (GR group) were patients who benefited from the treatment by achieving pCR corresponding to the histological disappearance of invasive cancer cells on breast tissue after surgery. Whilst patients who, after the neoadjuvant treatment, continued to show the presence of cancer cells in breast tissue were classified as poor responders (PR group).

The research of pre-treatment biomarkers predictive of the histological response was performed by analysing the quantitative differences in the serum targeted metabolomics profile among GR and PR group in a case-control study.

### Serum targeted metabolomics investigations

A high-throughput liquid chromatography tandem mass spectrometry (LC-MS/MS) platform has been applied to evaluate the serum metabolomics profile using the AbsoluteIDQ p180 Kit (Biocrates Life Sciences Innsbruck, Austria). The analytical system consisted of a liquid chromatography ultimate 3000 (Thermo Fisher Scientific Milan Italy) coupled with a 4000 Qtrap (ABsciex Framingham, MA, USA) mass spectrometer. The analytical method allows the quantification of 188 targeted metabolites covering the following compound classes: amino acids, biogenic amines and polyamines (*n* = 40), acylcarnitine (*n* = 40), di-acyl-phosphatidyl lipids (*n* = 92), sphingolipids (*n* = 15) and total exoses (*n* = 1). The complete list of the all metabolites investigated was reported in Table 2. Briefly, 10 μL of serum were loaded onto a inserted filter in a 96 well sandwich plate, which already contained appropriate internal standards structurally identical but labeled with stable isotopes such as deuterium, 13C, or 15N. The filters were dried in nitrogen stream, amino acids were derivatized with 5% phenylisothiocyanate reagent (PTC), and filters were dried again. After extraction of metabolites with 500 μL of 5 mM ammonium acetate in methanol, the solution was centrifuged through the filter membrane and diluted with water and MS running solvent. Final extracts were than analyzed by LC-MS/MS for amino acids and bioactive amines PTC-derivatives using a Zorbax SB 100 × 2.1 mm column (Agilent, Santa Clara CA, USA), while direct flow injection analysis (FIA-MS/MS) was used for the analysis of acylcarnitine and phospolipides. The specific signals of metabolites utilized for quantitation were achieved by multiple reaction monitoring, neutral loss and precursor ion scans in positive and negative ion mode. The MS/MS signals were integrated, by using Analyst 1.6.1(ABsciex Framingham, MA, USA) and concentrations obtained using a calibration curve according to the AS-180 manual. All quantitative data were further processed for verification and validation by the MetIQ software by comparing the results of triplicate analysis of low, medium and high quality serum controls as an integral part of the analytical kit. Within the analytical batch the coefficient of variation for the quality control metabolites concentration was less than 20%, while the uncertainty of the measurements was found in the range of 80–115% of their theoretical values. Samples were analyzed in a random sequence with serum controls in compliance with Want et al. 2010 guidelines [43]. The metabolites with serum concentration above the limit of detection in at least 50% of all measured samples were excluded by successive statistical analysis. Among the serum samples investigated, about 18% of analyzed metabolites showed serum concentrations below the lower limit of quantification criteria and were not considered for further data analysis. The final number of metabolites analyzed was 153 (82%). This set included 17 (42%) acylcarnitines, 14 (100%) lysophosphatidyl coline lipids, 72 (95%) phosphatidylcolines, 15 (100%) sphingomyelin lipids and 35 (83%) amino acids plus bioactive amine derivatives. The mean ± SD serum concentration of the validated metabolites among the GR and PR groups of patients are reported together with the statistical significance in supplemental data in Table S1

**Table 2:** List of metabolite determined using LC and FIA MS/MS platform

Metabolite	*N*		
Amino acids	21	Ala, Arg, Asn, Asp, Cit, Gln, Glu, Gly, His, Ile, Leu, Lys, Met, Orn, Phe, Pro, Ser, Thr, Trp, Tyr, Val	Amino acid metabolism, urea-cycle, activity of gluconeogenesis and glycolysis, insulin sensitivity, neurotransmitter metabolism, oxidative stress
Biogenic-amine	19	ADMA, AcOrn, *carnosine* (*), creatinine, *histamine*, kynurenine, Met-SO, *Nitro-Tyr*, *OH-Pro*, *PEA*, Putrescine, SDMA, sarcosine,serotonin, spermidine, *spermine*, taurine, alpha-AAA,totalDMA	Neurological disorders, cell proliferation, cell cycle progression, DNA stability, oxidative stress
Carnitine	1	C0	Energy metabolism, fatty acid transport and mitochondrial fatty acid oxidation, ketosis, oxidative stress, mitochondrial membrane damage
Acylcarnitines	26	C2, C3, C3:1, C4, C4:1, C5, C5:1, C6 (or C4:1-DC), C6:1, C8, C8:1, C9, C10, C10:1, C10:2, C12, C12:1,C14, C14:1, C14:2, C16, C16:1, C16:2, C18, C18:1,C18:2	“
Hydroxy- and dicarboxyacylcarnitines	13	C3-OH, C4-OH (or C3-DC), C5-DC (or C6-OH), C5-OH(or C3-DC-M), C5:1-DC, C5-M-DC, C7-DC, C12-DC, C14:1-OH, C14:2-OH, C16:1-OH, C16:2-OH, C16-OH,C18:1-OH	“
Sum of hexoses	1	H1	Carbohydrate metabolism
Sphingomyelins	10	SM C16:0, SM C16:1, SM C18:0, SM C18:1, SMC20:2, SM C22:3, SM C24:0, SMC24:1, SM C26:0,SM C26:1	Signalling cascades, membrane damage (eg,neurodegeneratio)n
Hydroxysphingomyelins	5	SM (OH) C14:1, SM (OH) C16:1, SM (OH) C22:1, SM(OH) C22:2, SM (OH) C24:1	“
Diacyl-phosphatidylcholines	38	PC aa C24:0/C26:0/C28:1/C30:0/C30:2/C32:0/C32:1/C32:2/C32:3/C34:1/C34:2/C34:3/C34:4/C36:0/C36:1/C36:2/C36:3/C36:4/C36:5/C36:6/C38:0C38:1/C38:3/C38:4/C38:5/C38:6/C40:1/C40:2C40:3/C40:4/C40:5/C40:6/C42:0/C42:1/C42:2/C42:4/C42:5/C42:6	Degradation of phospholipids, membrane damage, signalling cascades, fatty acid profile
Acyl-alkyl-phosphatidylcholines	39	PC ae C30:0/C30:1/C30:2/C32:1/C32:2/C34:0/C34:1/C34:2/C34:3/C36:0/C36:1/C36:2/C36:3/C36:4/C36:5/C38:0/C38:1/C38:2/C38:3/C38:4/C38:5/C38:6/C40:0/C40:1/C40:2/C40:3/C40:4/C40:5/C40:6/C42:0/C42:1/C42:2/C42:3/C42:4/C42:5/C44:3/C44:4/C44:5/C44:6	“
Lyso-phosphatidylcholines	15	lysoPC aa C6:0/C14:0/C16:0/C16:1/C17:0/C18:0/C18:1/C18:2/C20:3/C20:4/C24:0/C26:0/C26:1/C28:0/C28:1	Degradation of phospholipids (phospholipase activity), membrane damage, signaling cascades, fatty acid profiles

aa, acyl-acyl; ae, acyl-alkyl; a, lyso; Cx:y, where x is the number of carbons in the fatty acid side chain; y is the number of double bonds in the fatty acid side chain; DC, decarboxyl; M,methyl; OH, hydroxyl; PC, phophatidylcholine; SM, sphingomyelin. (*) *Underlined* the metabolites that did not pass the analytical quality control process.

### Data processing and statistical analysis

Prior to statistical analysis, the concentration values of metabolites were set to a log scale and auto-scaled (mean-centred and divided by the standard deviation of each variable). A supervisor multivariate partial least squares discrimination analysis (PLS-DA) was then applied to identify the relevant metabolites that contribute the most significantly in differentiating between the two groups of patients. The PLS model was refined by incorporating only the metabolite variable that was identified as being significantly different among the two groups of patients (*p* < 0.05). A variable importance in projection (VIP) score was also applied to rank the patient's metabolites that best distinguished between the GR *vs* PR group. The PSA-DA model was further cross-validated by comparison of the resulting goodness of fit (*R*^2^), predictive ability (*Q*^2^) values, and by internal validation using 1000 permutation tests. *Q*^2^ represents the most recognized diagnostic statistical parameter to validate the PLS-DA model in metabolomics. It is calculated as 1-PRESS/TSS where PRESS is the sum of squared differences between the predicted and real value, while the TSS is the total sum of the square. Significant predictive model is considered for *Q*^2^ value greater than 0.4. *R*^2^ indicates the total variation in the data matrix that is explained by the model. It is calculated as 1- RSS/TSS where the RSS is the fitted residual sum of the square of the data. An invalid or irrelevant model may be still capable of producing a large *Q*^2^ value, because consistent cross-validation requires a systematic deletion of large portions of the dataset during training. Thus, further validation of the model was performed by random 1000 permutation of class labels, which requires no deletion of data, with internal leave-n-out cross-validation. Significance of permutation test was expressed as pseudo p calculated as number of failed prediction / numbers of permutation performed.

The significant features were further selected by taking into account the problem of false discovery rate (FDR) occurring for multiple analyses [44], through the application of the significance analysis of microarray (SAM) approach. An un-paired *t*-test was used as a univariate analysis to check the differences in the serum concentrations of single metabolites between the GR and the PR groups. For all the above univariate tests *q* values were calculated to control for multiple testing false discovery rate.

Receiver operating characteristic (ROC) curves analysis was used to determine the diagnostic power of the more significant metabolomics biomarkers. In a ROC curve, the true positive rate (sensitivity) was plotted against the false positive rate (1-specificity) for different cut-off points of a given parameter. The ROC curve was validated by Internal cross validation and permutation testing. The optimal cut-off was assessed by jointly maximizing sensitivity and specificity. Sensitivity and specificity, computed at the optimal cut-off, were then used for further investigation. All above data processing and the statistical analysis that included ROC analysis was performed using the Metabolanalyst web portals [45, 46].

## SUPPLEMENTARY MATERIALS FIGURE AND TABLE




