# Supplementary material for: Pharmacometabolomics study identifies circulating spermidine and tryptophan as potential biomarkers associated with the complete pathological response to trastuzumab-paclitaxel neoadjuvant therapy in HER-2 positive breast cancer
Source: Oncotarget. 2016 May 19;7(26):39809–22. doi: 10.18632/oncotarget.9489 (PMC5129972; doi:10.18632/oncotarget.9489)
Supplement: Supplementary file 1 [file oncotarget-07-39809-s001.pdf]

Pharmacometabolomics study identifies circulating spermidine and tryptophan as potential biomarkers associated with the complete pathological response to trastuzumab-paclitaxel neoadjuvant therapy in HER-2 positive breast cancer

Supplementary Materials

SUMMARY

This file contains additional data regarding the serum concentration of the metabolites investigated for the GR and PR groups of patients not full reported in the main manuscript and the effect that the confounding factors such as Age BMI and stage of disease have on the diagnostic power of the predictive model based on the pre- treatment serum level of tryptophan and spermidine.

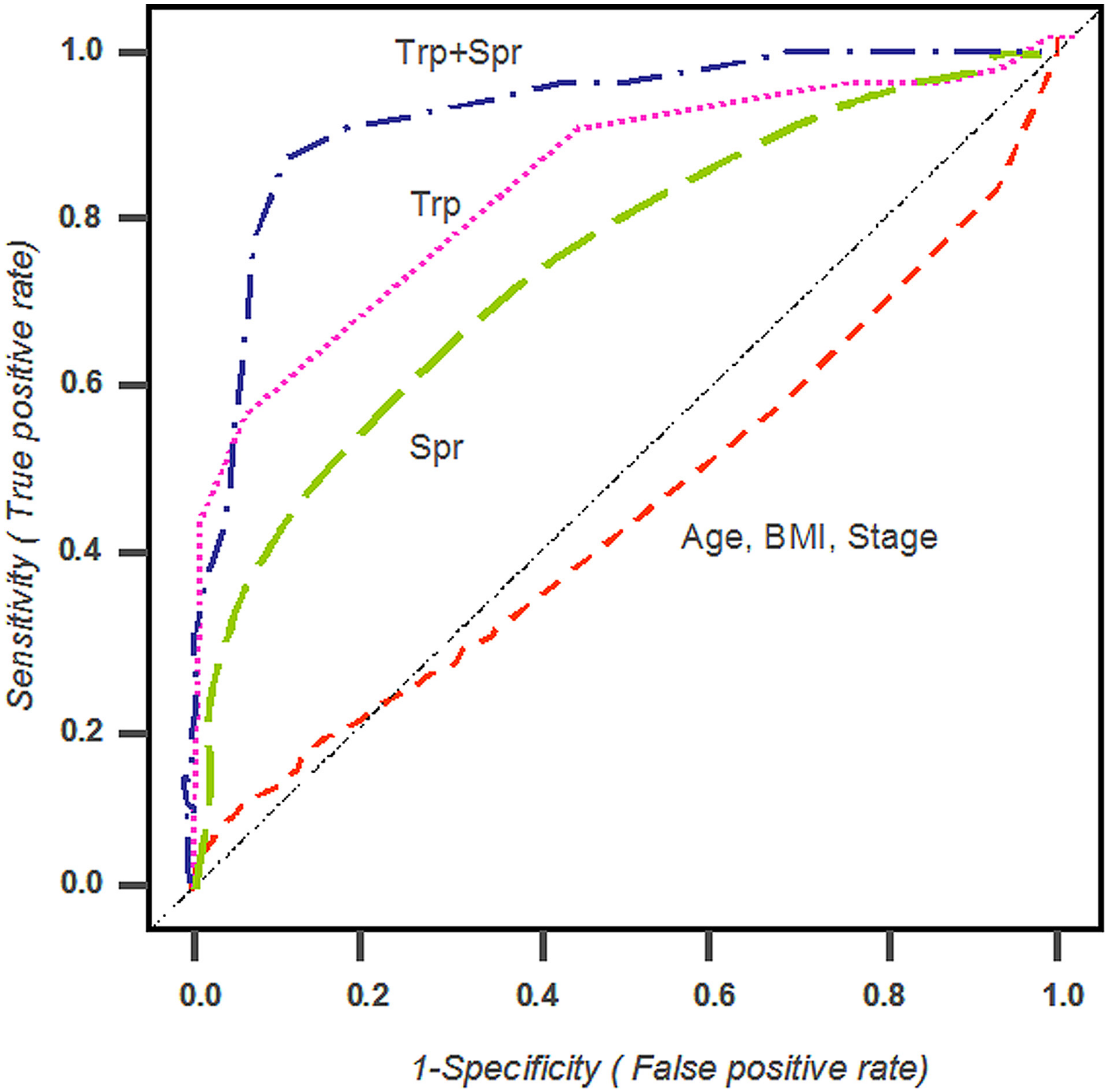

Figure 1: Relative contribution of metabolites Trp and Spd and covariates: age, BMI and stage disease in the discrimination of GR and PR patients to trastuzumab-paclitaxel neoadjuvant treatment. The receiver operating characteristic (ROC) curves indicated the effect of discrimination based on the combination of age, sex and BMI (red) AUC = 045, Spd (green) AUC = 0.83, Trp (pink) AUC = 0.87 and Trp and Spd (blue) AUC = 0.93.

Supplementary Table S1: Metabolites serum concentration in GR and PR groups of patients. See Supplementary_Table_S1
