# Supplementary material for: Pharmacometabolomics study identifies circulating spermidine and tryptophan as potential biomarkers associated with the complete pathological response to trastuzumab-paclitaxel neoadjuvant therapy in HER-2 positive breast cancer
Source: Oncotarget. 2016 May 19;7(26):39809–22. doi: 10.18632/oncotarget.9489 (PMC5129972; doi:10.18632/oncotarget.9489)
Supplement: Supplementary file 2 [file oncotarget-07-39809-s002.docx]

**Supplementary Table S1: Metabolites serum concentration in GR and PR groups of patients**

| **PR** | | | | | **GR** | | | | | |
| --- | --- | --- | --- | --- | --- | --- | --- | --- | --- | --- |
| **Meatabolite** | **Mean** | **SD** | **Min** | **Max** | **Mean** | **SD** | **Min** | **Max** | ***p* value*** | ***q* value#** |
| ***C0*** | ***41.38*** | ***8.08*** | ***21.23*** | ***52.60*** | ***34.33*** | ***9.06*** | ***20.75*** | ***47.69*** | ***0.028614*** | ***0.32293*** |
| C10 | 0.34 | 0.18 | 0.10 | 0.84 | 0.30 | 0.12 | 0.15 | 0.61 | 0.71626 | 0.95737 |
| C10:2 | 0.06 | 0.01 | 0.03 | 0.08 | 0.06 | 0.01 | 0.05 | 0.087 | 0.56338 | 0.87917 |
| C14:1 | 0.22 | 0.06 | 0.12 | 0.35 | 0.22 | 0.06 | 0.12 | 0.361 | 0.96877 | 0.99811 |
| C14:2 | 0.04 | 0.02 | 0.01 | 0.09 | 0.04 | 0.01 | 0.02 | 0.056 | 0.80381 | 0.96735 |
| C16 | 0.12 | 0.04 | 0.06 | 0.21 | 0.10 | 0.02 | 0.07 | 0.13 | 0.25348 | 0.77019 |
| C18:1 | 0.13 | 0.04 | 0.07 | 0.21 | 0.12 | 0.04 | 0.07 | 0.21 | 0.76272 | 0.95737 |
| C18:2 | 0.05 | 0.02 | 0.02 | 0.13 | 0.04 | 0.01 | 0.03 | 0.058 | 0.29721 | 0.77864 |
| C2 | 6.54 | 3.20 | 3.24 | 16.27 | 5.93 | 2.45 | 3.36 | 9.91 | 0.567 | 0.87917 |
| ***C3*** | ***0.38*** | ***0.13*** | ***0.17*** | ***0.79*** | ***0.28*** | ***0.07*** | ***0.17*** | ***0.38*** | ***0.0095876*** | ***0.28436*** |
| C3–DC (C4–OH) | 0.04 | 0.03 | 0.01 | 0.14 | 0.03 | 0.01 | 0.02 | 0.043 | 0.38944 | 0.80513 |
| C4 | 0.19 | 0.05 | 0.10 | 0.28 | 0.17 | 0.05 | 0.10 | 0.32 | 0.20725 | 0.71185 |
| C5 | 0.11 | 0.03 | 0.05 | 0.16 | 0.10 | 0.02 | 0.07 | 0.12 | 0.33686 | 0.80513 |
| C5-DC (C6-OH) | 0.02 | 0.01 | 0.01 | 0.03 | 0.02 | 0.01 | 0.01 | 0.031 | 0.9831 | 0.99959 |
| C6 (C4:1-DC) | 0.07 | 0.03 | 0.03 | 0.15 | 0.06 | 0.02 | 0.04 | 0.10 | 0.42804 | 0.80513 |
| C8 | 0.25 | 0.15 | 0.09 | 0.72 | 0.23 | 0.09 | 0.13 | 0.42 | 0.93479 | 0.99811 |
| lysoPC a C14:0 | 4.66 | 0.88 | 3.10 | 6.87 | 4.44 | 0.92 | 3.08 | 6.32 | 0.44479 | 0.80779 |
| lysoPC a C16:0 | 166.78 | 38.01 | 90.97 | 244.91 | 151.52 | 24.45 | 104.78 | 203.03 | 0.27778 | 0.77291 |
| lysoPC a C16:1 | 4.87 | 1.43 | 1.91 | 8.26 | 4.59 | 1.30 | 3.21 | 7.29 | 0.68504 | 0.95185 |
| lysoPC a C17:0 | 3.37 | 1.02 | 1.72 | 5.63 | 3.39 | 0.68 | 2.66 | 4.72 | 0.73396 | 0.95737 |
| lysoPC a C18:0 | 50.77 | 16.22 | 26.07 | 103.53 | 46.41 | 6.56 | 36.81 | 55.97 | 0.49771 | 0.84557 |
| lysoPC a C18:1 | 33.61 | 8.67 | 14.95 | 49.09 | 33.63 | 5.43 | 28.56 | 47.42 | 0.75583 | 0.95737 |
| lysoPC a C18:2 | 41.58 | 13.76 | 15.28 | 66.24 | 40.35 | 9.49 | 25.16 | 55.62 | 0.97284 | 0.99811 |
| lysoPC a C20:3 | 4.07 | 1.16 | 1.93 | 6.77 | 3.54 | 0.99 | 2.21 | 6.13 | 0.19163 | 0.69056 |
| lysoPC a C20:4 | 11.82 | 3.35 | 6.38 | 17.37 | 10.35 | 2.35 | 6.26 | 15.18 | 0.23527 | 0.76067 |
| lysoPC a C24:0 | 0.61 | 0.26 | 0.38 | 1.48 | 0.54 | 0.11 | 0.39 | 0.81 | 0.37842 | 0.80513 |
| ***lysoPC a C26:0*** | ***0.58*** | ***0.46*** | ***0.22*** | ***2.01*** | ***0.32*** | ***0.17*** | ***0.15*** | ***0.83*** | ***0.012286*** | ***0.28436*** |
| ***lysoPC a C26:1*** | ***0.52*** | ***0.47*** | ***0.19*** | ***2.00*** | ***0.28*** | ***0.12*** | ***0.13*** | ***0.64*** | ***0.016051*** | ***0.31701*** |
| ***lysoPC a C28:0*** | ***0.56*** | ***0.39*** | ***0.23*** | ***1.62*** | ***0.37*** | ***0.16*** | ***0.20*** | ***0.85*** | ***0.047103*** | ***0.38443*** |
| ***lysoPC a C28:1*** | ***0.85*** | ***0.47*** | ***0.42*** | ***2.14*** | ***0.59*** | ***0.18*** | ***0.30*** | ***1.09*** | ***0.02432*** | ***0.32293*** |
| ***PC aa C24:0*** | ***0.22*** | ***0.17*** | ***0.07*** | ***0.71*** | ***0.12*** | ***0.06*** | ***0.08*** | ***0.31*** | ***0.012598*** | ***0.28436*** |
| ***PC aa C26:0*** | ***1.40*** | ***0.78*** | ***0.76*** | ***3.52*** | ***0.94*** | ***0.33*** | ***0.61*** | ***1.98*** | ***0.010865*** | ***0.28436*** |
| PC aa C28:1 | 4.10 | 0.87 | 2.84 | 5.84 | 3.90 | 0.89 | 2.56 | 5.52 | 0.46792 | 0.82145 |
| PC aa C30:0 | 6.15 | 1.18 | 4.25 | 8.39 | 5.93 | 1.16 | 4.26 | 8.29 | 0.57705 | 0.87917 |
| ***PC aa C30:2*** | ***1.64*** | ***1.20*** | ***0.39*** | ***5.33*** | ***2.57*** | ***1.27*** | ***0.91*** | ***4.21*** | ***0.025326*** | ***0.32293*** |
| PC aa C32:0 | 14.57 | 3.50 | 10.61 | 25.67 | 13.00 | 2.03 | 9.64 | 16.38 | 0.12552 | 0.56663 |
| PC aa C32:1 | 17.66 | 8.04 | 6.28 | 36.43 | 14.04 | 6.05 | 5.19 | 29.05 | 0.15606 | 0.60763 |
| PC aa C32:2 | 4.35 | 1.33 | 2.22 | 7.39 | 3.99 | 1.32 | 2.33 | 6.71 | 0.42503 | 0.80513 |
| PC aa C32:3 | 0.60 | 0.08 | 0.47 | 0.76 | 0.61 | 0.15 | 0.41 | 1.03 | 0.87826 | 0.96735 |
| PC aa C34:1 | 187.85 | 30.21 | 122.43 | 241.78 | 172.02 | 31.59 | 115.80 | 230.72 | 0.1477 | 0.60763 |
| PC aa C34:2 | 255.96 | 41.11 | 192.71 | 339.17 | 234.93 | 26.91 | 199.24 | 290.36 | 0.10726 | 0.55182 |
| PC aa C34:3 | 15.06 | 4.08 | 8.55 | 24.12 | 12.91 | 3.63 | 7.53 | 21.768 | 0.10939 | 0.55182 |
| PC aa C34:4 | 2.21 | 0.53 | 1.32 | 3.25 | 1.93 | 0.68 | 1.08 | 3.715 | 0.10585 | 0.55182 |
| PC aa C36:0 | 3.04 | 0.90 | 1.79 | 4.92 | 3.09 | 0.78 | 1.87 | 4.80 | 0.7805 | 0.96344 |
| PC aa C36:1 | 57.20 | 9.79 | 36.38 | 74.45 | 55.29 | 12.91 | 30.81 | 74.52 | 0.53139 | 0.86557 |
| PC aa C36:2 | 180.40 | 32.94 | 122.14 | 262.26 | 167.67 | 25.76 | 124.05 | 202.7 | 0.2406 | 0.76067 |
| ***PC aa C36:3*** | ***123.20*** | ***21.45*** | ***86.77*** | ***171.33*** | ***107.09*** | ***22.93*** | ***61.42*** | ***138.87*** | ***0.040476*** | ***0.38443*** |
| ***PC aa C36:4*** | ***165.89*** | ***33.09*** | ***121.35*** | ***273.06*** | ***138.03*** | ***26.47*** | ***87.20*** | ***189.68*** | ***0.0080222*** | ***0.28436*** |
| PC aa C36:5 | 19.98 | 9.28 | 10.41 | 47.15 | 20.44 | 11.11 | 9.23 | 46.431 | 0.9616 | 0.99811 |
| PC aa C36:6 | 1.20 | 0.40 | 0.61 | 2.01 | 1.36 | 0.57 | 0.61 | 2.501 | 0.42588 | 0.80513 |
| ***PC aa C38:3*** | ***64.26*** | ***12.19*** | ***38.98*** | ***86.79*** | ***55.36*** | ***12.60*** | ***31.84*** | ***78.619*** | ***0.042625*** | ***0.38443*** |
| PC aa C38:4 | 117.57 | 24.73 | 91.35 | 190.22 | 99.55 | 20.60 | 70.17 | 140.43 | 0.020764 | 0.32293 |
| PC aa C38:5 | 56.38 | 9.36 | 35.68 | 77.43 | 53.74 | 13.75 | 31.35 | 80.15 | 0.38635 | 0.80513 |
| PC aa C40:1 | 0.41 | 0.08 | 0.29 | 0.56 | 0.42 | 0.08 | 0.27 | 0.56 | 0.7627 | 0.95737 |
| PC aa C40:2 | 0.37 | 0.09 | 0.21 | 0.49 | 0.35 | 0.09 | 0.21 | 0.59 | 0.51668 | 0.86373 |
| PC aa C40:3 | 0.60 | 0.11 | 0.44 | 0.91 | 0.58 | 0.16 | 0.38 | 1.00 | 0.42383 | 0.80513 |
| PC aa C40:4 | 4.71 | 1.55 | 2.86 | 8.70 | 3.84 | 1.02 | 2.32 | 6.36 | 0.063988 | 0.38986 |
| PC aa C40:5 | 10.98 | 2.62 | 5.97 | 16.52 | 9.96 | 2.55 | 5.63 | 15.15 | 0.24591 | 0.76185 |
| PC aa C40:6 | 30.72 | 10.54 | 17.05 | 55.35 | 30.47 | 6.64 | 21.35 | 45.18 | 0.81796 | 0.96735 |
| PC aa C42:0 | 0.72 | 0.22 | 0.41 | 1.20 | 0.79 | 0.25 | 0.45 | 1.51 | 0.3243 | 0.80513 |
| PC aa C42:1 | 0.34 | 0.09 | 0.19 | 0.59 | 0.38 | 0.12 | 0.17 | 0.60 | 0.38509 | 0.80513 |
| PC aa C42:2 | 0.28 | 0.08 | 0.15 | 0.43 | 0.30 | 0.06 | 0.20 | 0.41 | 0.43812 | 0.80779 |
| PC aa C42:4 | 0.26 | 0.06 | 0.17 | 0.35 | 0.24 | 0.05 | 0.15 | 0.30 | 0.44097 | 0.80779 |
| PC aa C42:6 | 0.81 | 0.13 | 0.60 | 1.06 | 0.80 | 0.11 | 0.63 | 1.08 | 0.95264 | 0.99811 |
| PC ae C30:0 | 0.40 | 0.08 | 0.23 | 0.53 | 0.39 | 0.09 | 0.27 | 0.55 | 0.853 | 0.96735 |
| PC ae C30:1 | 0.50 | 0.22 | 0.18 | 1.06 | 0.38 | 0.16 | 0.22 | 0.70 | 0.0746 | 0.43655 |
| PC ae C30:2 | 0.34 | 0.08 | 0.23 | 0.51 | 0.29 | 0.11 | 0.18 | 0.58 | 0.051163 | 0.38443 |
| PC ae C32:1 | 3.03 | 0.59 | 1.83 | 4.30 | 3.13 | 0.66 | 1.99 | 4.26 | 0.6681 | 0.9425 |
| PC ae C32:2 | 0.88 | 0.12 | 0.68 | 1.13 | 0.87 | 0.24 | 0.52 | 1.38 | 0.6004 | 0.87917 |
| PC ae C34:0 | 1.55 | 0.21 | 1.15 | 2.01 | 1.47 | 0.39 | 0.81 | 2.49 | 0.27792 | 0.77291 |
| PC ae C34:1 | 10.46 | 1.63 | 7.63 | 13.49 | 10.77 | 2.40 | 6.81 | 15.04 | 0.80558 | 0.96735 |
| PC ae C34:2 | 11.43 | 3.83 | 5.91 | 20.34 | 11.18 | 1.96 | 7.25 | 14.70 | 0.87614 | 0.96735 |
| PC ae C34:3 | 8.76 | 3.17 | 4.47 | 14.36 | 7.97 | 1.33 | 5.85 | 9.95 | 0.662 | 0.9423 |
| PC ae C36:0 | 0.72 | 0.18 | 0.43 | 1.11 | 0.66 | 0.13 | 0.49 | 1.09 | 0.37116 | 0.80513 |
| PC ae C36:1 | 9.92 | 1.28 | 7.36 | 11.88 | 9.81 | 2.70 | 5.97 | 15.59 | 0.60095 | 0.87917 |
| PC ae C36:2 | 15.02 | 3.00 | 10.31 | 21.93 | 14.86 | 2.97 | 10.39 | 21.68 | 0.88433 | 0.96735 |
| PC ae C36:3 | 8.39 | 2.36 | 5.33 | 13.89 | 8.10 | 1.81 | 5.06 | 11.04 | 0.76849 | 0.95737 |
| PC ae C36:4 | 19.29 | 4.64 | 13.13 | 32.43 | 17.94 | 3.97 | 10.50 | 22.98 | 0.35095 | 0.80513 |
| PC ae C36:5 | 14.22 | 3.61 | 9.96 | 22.06 | 13.32 | 2.83 | 8.58 | 18.99 | 0.46213 | 0.82041 |
| PC ae C38:0 | 1.84 | 0.39 | 1.31 | 2.58 | 2.05 | 0.67 | 1.28 | 3.77 | 0.32944 | 0.80513 |
| ***PC ae C3S:1*** | ***1.04*** | ***0.55*** | ***0.03*** | ***2.25*** | ***0.S5*** | ***0.36*** | ***0.07*** | ***1.29*** | ***0.0493SS*** | ***0.3S443*** |
| PC ae C38:2 | 2.23 | 0.38 | 1.50 | 3.01 | 2.34 | 0.75 | 1.22 | 3.68 | 0.88102 | 0.96735 |
| PC ae C38:3 | 4.69 | 0.67 | 3.85 | 5.95 | 4.33 | 1.02 | 2.58 | 5.93 | 0.15748 | 0.60763 |
| PC ae C38:4 | 16.17 | 1.98 | 13.45 | 19.90 | 16.15 | 3.56 | 8.96 | 21.31 | 0.75382 | 0.95737 |
| PC ae C38:5 | 21.63 | 4.42 | 15.09 | 32.99 | 21.15 | 5.00 | 13.18 | 27.79 | 0.68678 | 0.95185 |
| PC ae C38:6 | 8.98 | 2.49 | 5.62 | 15.70 | 9.38 | 2.47 | 5.79 | 16.30 | 0.59735 | 0.87917 |
| PC ae C40:1 | 1.27 | 0.32 | 0.75 | 1.77 | 1.23 | 0.27 | 0.87 | 1.89 | 0.76953 | 0.95737 |
| PC ae C40:2 | 2.48 | 0.56 | 2.02 | 3.99 | 2.46 | 0.56 | 1.61 | 3.73 | 0.86819 | 0.96735 |
| PC ae C40:3 | 1.31 | 0.16 | 1.09 | 1.63 | 1.34 | 0.16 | 1.03 | 1.60 | 0.56583 | 0.87917 |
| PC ae C40:4 | 2.45 | 0.46 | 1.81 | 3.64 | 2.50 | 0.44 | 1.66 | 3.17 | 0.73189 | 0.95737 |
| PC ae C40:5 | 4.28 | 0.70 | 3.13 | 5.62 | 4.64 | 0.99 | 3.32 | 6.25 | 0.27883 | 0.77291 |
| PC ae C40:6 | 5.37 | 1.29 | 3.51 | 7.74 | 6.15 | 1.26 | 4.51 | 9.33 | 0.064154 | 0.38986 |
| PC ae C42:0 | 0.51 | 0.09 | 0.37 | 0.67 | 0.47 | 0.07 | 0.37 | 0.63 | 0.19231 | 0.69056 |
| ***PC ae C42:1*** | ***0.40*** | ***0.0S*** | ***0.29*** | ***0.55*** | ***0.34*** | ***0.0S*** | ***0.27*** | ***0.52*** | ***0.027264*** | ***0.32293*** |
| PC ae C42:2 | 0.67 | 0.10 | 0.50 | 0.85 | 0.65 | 0.13 | 0.46 | 1.01 | 0.5858 | 0.87917 |
| PC ae C42:3 | 0.87 | 0.16 | 0.60 | 1.25 | 0.90 | 0.18 | 0.62 | 1.35 | 0.61212 | 0.8873 |
| PC ae C42:4 | 0.89 | 0.23 | 0.55 | 1.35 | 0.92 | 0.18 | 0.66 | 1.25 | 0.58031 | 0.87917 |
| PC ae C42:5 | 2.95 | 0.59 | 2.09 | 4.00 | 3.26 | 0.56 | 2.39 | 4.23 | 0.11623 | 0.55182 |
| PC ae C44:3 | 0.17 | 0.04 | 0.10 | 0.23 | 0.15 | 0.03 | 0.11 | 0.19 | 0.41697 | 0.80513 |
| PC ae C44:4 | 0.52 | 0.13 | 0.32 | 0.95 | 0.53 | 0.08 | 0.40 | 0.65 | 0.69983 | 0.95737 |
| PC ae C44:5 | 2.57 | 0.74 | 1.51 | 3.92 | 2.82 | 0.74 | 1.84 | 4.28 | 0.28638 | 0.77864 |
| PC ae C44:6 | 1.67 | 0.68 | 0.98 | 3.92 | 1.77 | 0.48 | 1.16 | 2.95 | 0.39793 | 0.80513 |
| SM (OH) C14:1 | 7.46 | 1.94 | 4.71 | 13.49 | 7.49 | 1.87 | 4.26 | 11.818 | 0.96904 | 0.99811 |
| SM (OH) C16:1 | 4.83 | 1.13 | 3.09 | 7.86 | 4.88 | 1.05 | 3.29 | 7.46 | 0.83993 | 0.96735 |
| SM (OH) C22:1 | 19.38 | 4.10 | 12.02 | 26.67 | 19.46 | 3.06 | 14.23 | 26.31 | 0.84006 | 0.96735 |
| SM (OH) C22:2 | 15.40 | 2.92 | 11.93 | 24.01 | 15.03 | 3.23 | 11.01 | 22.15 | 0.66192 | 0.9423 |
| SM (OH) C24:1 | 2.29 | 0.40 | 1.47 | 3.16 | 2.27 | 0.36 | 1.51 | 3.08 | 0.94453 | 0.99811 |
| SM C16:0 | 130.97 | 24.47 | 87.04 | 180.04 | 127.01 | 14.11 | 99.27 | 143.70 | 0.70592 | 0.95737 |
| SM C16:1 | 23.18 | 5.01 | 16.71 | 38.01 | 20.65 | 4.17 | 15.93 | 27.74 | 0.098645 | 0.55182 |
| SM C18:0 | 30.42 | 5.98 | 20.12 | 41.32 | 30.26 | 5.17 | 24.02 | 40.02 | 0.99799 | 0.99959 |
| SM C18:1 | 15.47 | 3.24 | 11.00 | 21.55 | 14.64 | 3.68 | 10.56 | 21.11 | 0.40537 | 0.80513 |
| SM C20:2 | 0.89 | 0.43 | 0.31 | 1.75 | 0.82 | 0.28 | 0.36 | 1.38 | 0.93561 | 0.99811 |
| SM C22:3 | 3.95 | 3.63 | 0.40 | 10.77 | 3.78 | 2.85 | 0.65 | 8.68 | 0.80862 | 0.96735 |
| SM C24:0 | 34.69 | 8.37 | 20.22 | 49.65 | 33.68 | 3.79 | 24.69 | 40.03 | 0.91036 | 0.98518 |
| SM C24:1 | 65.97 | 11.24 | 41.60 | 85.74 | 65.30 | 11.70 | 45.42 | 88.84 | 0.86848 | 0.96735 |
| SM C26:0 | 0.28 | 0.08 | 0.12 | 0.42 | 0.29 | 0.05 | 0.21 | 0.38 | 0.42304 | 0.80513 |
| SM C26:1 | 0.61 | 0.15 | 0.29 | 0.81 | 0.62 | 0.13 | 0.48 | 0.85 | 0.7679 | 0.95737 |
| H1 | 5799.78 | 2011.55 | 2915.94 | 11704.30 | 4881.53 | 649.89 | 4047.38 | 6437.4 | 0.13778 | 0.58837 |
| Ala | 432.26 | 90.62 | 263.00 | 576.00 | 382.00 | 73.88 | 232.00 | 502.00 | 0.11875 | 0.55182 |
| Arg | 106.38 | 23.54 | 59.50 | 155.00 | 106.10 | 30.20 | 48.40 | 154.00 | 0.8338 | 0.078939 |
| Asn | 57.67 | 10.99 | 40.00 | 82.20 | 53.41 | 5.84 | 45.10 | 67.70 | 0.24072 | 0.61849 |
| Asp | 27.30 | 8.09 | 14.80 | 43.10 | 25.39 | 6.62 | 17.60 | 37.80 | 0.54752 | 0.2616 |
| Cit | 30.43 | 7.72 | 22.40 | 50.80 | 27.45 | 5.66 | 16.20 | 36.80 | 0.21645 | 0.72763 |
| Gln | 582.37 | 89.82 | 446.00 | 821.00 | 555.07 | 58.58 | 471.00 | 641.00 | 0.35729 | 0.80513 |
| Glu | 86.46 | 33.55 | 41.50 | 160.00 | 78.07 | 36.00 | 30.10 | 149.00 | 0.41575 | 0.80513 |
| Gly | 496.26 | 204.46 | 261.00 | 1110.00 | 472.80 | 134.29 | 295.00 | 786.00 | 0.88775 | 0.96735 |
| His | 86.63 | 11.14 | 67.10 | 117.00 | 79.21 | 11.74 | 57.80 | 99.20 | 0.062367 | 0.38986 |
| ***Ile*** | ***84.99*** | ***17.51*** | ***55.40*** | ***121.00*** | ***73.27*** | ***14.80*** | ***52.90*** | ***103.00*** | ***0.039687*** | ***0.38443*** |
| Leu | 184.42 | 39.77 | 106.00 | 274.00 | 158.80 | 30.17 | 109.00 | 226.00 | 0.050945 | 0.38443 |
| Lys | 207.37 | 30.99 | 144.00 | 259.00 | 187.07 | 25.21 | 146.00 | 245.00 | 0.053529 | 0.38443 |
| Met | 21.24 | 5.18 | 12.70 | 34.20 | 18.87 | 4.08 | 11.80 | 24.90 | 0.15768 | 0.60763 |
| Orn | 86.75 | 22.40 | 57.20 | 136.00 | 80.90 | 15.85 | 62.60 | 121.00 | 0.46177 | 0.82041 |
| Phe | 82.70 | 12.66 | 61.40 | 110.00 | 76.15 | 13.51 | 53.00 | 103.00 | 0.13423 | 0.58837 |
| Pro | 223.89 | 47.54 | 155.00 | 317.00 | 236.93 | 59.55 | 141.00 | 341.00 | 0.57985 | 0.87917 |
| Ser | 173.68 | 34.25 | 112.00 | 246.00 | 165.20 | 33.05 | 126.00 | 241.00 | 0.47385 | 0.82273 |
| Thr | 138.67 | 31.87 | 85.80 | 212.00 | 148.33 | 33.80 | 107.00 | 203.0 | 0.39089 | 0.80513 |
| ***Trp*** | ***73.82*** | ***9.23*** | ***53.00*** | ***91.60*** | ***61.19*** | ***8.46*** | ***38.30*** | ***72.0*** | ***0.00044585*** | ***0.035222*** |
| Tyr | 76.27 | 15.01 | 47.70 | 118.00 | 67.36 | 10.29 | 53.90 | 87.8 | 0.062143 | 0.38986 |
| Val | 343.37 | 97.01 | 214.00 | 526.00 | 301.93 | 67.30 | 195.00 | 458.00 | 0.1872 | 0.69056 |
| Ac-Orn | 0.86 | 0.47 | 0.29 | 2.07 | 0.82 | 0.35 | 0.35 | 1.51 | 0.99438 | 0.99959 |
| ADMA | 0.55 | 0.06 | 0.42 | 0.68 | 0.53 | 0.06 | 0.43 | 0.64 | 0.52945 | 0.86557 |
| SDMA | 0.36 | 0.18 | 0.15 | 0.73 | 0.30 | 0.16 | 0.05 | 0.61r | 0.86175 | 0.96735 |
| total DMA | 1.04 | 0.17 | 0.76 | 1.39 | 1.02 | 0.14 | 0.82 | 1.31 | 0.71387 | 0.95737 |
| alpha-AAA | 1.12 | 0.17 | 0.81 | 1.38 | 1.02 | 0.25 | 0.74 | 1.55 | 0.11329 | 0.55182 |
| Creatinine | 80.12 | 11.60 | 59.20 | 102.00 | 76.05 | 10.20 | 63.40 | 98.2 | 0.29955 | 0.77864 |
| Histamine | 0.48 | 0.00 | 0.48 | 0.49 | 0.48 | 0.00 | 0.48 | 0.49 | 0.3258 | 0.80513 |
| ***Kynurenine*** | ***2.80*** | ***0.71*** | ***1.77*** | ***4.51*** | ***2.29*** | ***0.35*** | ***1.58*** | ***3.18*** | ***0.018308*** | ***0.32142*** |
| Met-SO | 0.78 | 0.20 | 0.55 | 1.19 | 0.74 | 0.16 | 0.56 | 1.05 | 0.49025 | 0.84195 |
| Putrescine | 0.13 | 0.05 | 0.07 | 0.26 | 0.14 | 0.06 | 0.07 | 0.32 | 0.51933 | 0.86373 |
| Sarcosine | 22.70 | 4.82 | 14.20 | 30.00 | 20.79 | 3.49 | 13.30 | 25.6 | 0.27781 | 0.77291 |
| Serotonin | 0.55 | 0.27 | 0.22 | 1.33 | 0.60 | 0.16 | 0.38 | 0.99 | 0.30061 | 0.77864 |
| ***Spermidine*** | ***0.09*** | ***0.03*** | ***0.05*** | ***0.16*** | ***0.15*** | ***0.06*** | ***0.08*** | ***0.30*** | ***0.00044291*** | ***0.035222*** |
| Taurine | 153.47 | 28.01 | 112.00 | 215.00 | 167.6 | 34.37 | 115.00 | 241.00 | 0.20495 | 0.71185 |

**p* value for *T*-test for unpaired data, # adjusted *p*-values using optimised FDR approach for multiple testing.
